# DNA Encapsidation and Capsid Assembly Are Underexploited Antiviral Targets for the Treatment of Herpesviruses

**DOI:** 10.3389/fmicb.2020.01862

**Published:** 2020-08-12

**Authors:** Tara Keil, Dongmei Liu, Megan Lloyd, Wanda Coombs, Jennifer Moffat, Robert Visalli

**Affiliations:** ^1^Department of Biomedical Sciences, Mercer University School of Medicine, Savannah, GA, United States; ^2^Department of Microbiology and Immunology, Upstate Medical University, Syracuse, NY, United States

**Keywords:** antiviral, herpesvirus, letermovir, portal, terminase, encapsidation

## Abstract

Although there are effective nucleoside analogs to treat HSV, VZV, and HCMV disease, herpesvirus infections continue to contribute to significant morbidity and mortality. Acyclovir is the drug of choice for HSV encephalopathy, yet there is an estimated 6–19% mortality rate with half of the survivors experiencing moderate to severe chronic neurological deficits. For VZV, current treatments are inadequate to prevent acute and persistent pain due to zoster. Treatment of HCMV with GCV requires close monitoring particularly in patients with impaired renal function and there are no approved treatments for congenital HCMV infections. New therapeutic options to control cytomegalovirus reactivation in bone marrow and stem cell transplant patients are needed to improve patient outcome. No successful chemotherapeutic options are available for EBV, HHV-6, 7, and 8. Drug resistance is a concern for HCMV, HSV, and VZV since approved drugs share common mechanisms of action. Targeting DNA encapsidation or capsid assembly provide additional options for the development of non-nucleoside, small molecule anti-herpesviral drugs.

## Human Herpesviral Diseases – Toward Reducing Worldwide Morbidity and Mortality

The enveloped, dsDNA human herpesviruses (*Herpesviridae*) are classified as either alpha, beta or gamma herpesviruses depending on their genetic relatedness, host range, replication cycle and latency properties (Arvin, 2007). The subfamily Alphaherpesvirinae includes three relatively ubiquitous pathogens, herpes simplex viruses type 1 (HSV-1) and type 2 (HSV-2), and varicella-zoster virus (VZV). All three infect mucosal epithelia and establish life-long latency in cells of neuronal origin.

Primary infection and reactivation of latent HSV-1 or 2 results in asymptomatic shedding from the originally infected mucosal surface and/or symptomatic disease in the form of herpes labialis (cold sores) or genital lesions. Worldwide, 3.7 billion people under age 50 (67%) and 417 million people aged 15–49 (11%) are estimated to be infected with HSV-1 and HSV-2, respectively (Looker et al., 2015a,b). Genital herpes caused by either HSV-1 or HSV-2 is estimated to affect >400 million persons worldwide (Groves, 2016). HSV-1 is primarily associated with herpes labialis and HSV-2 with epidemic sexually transmitted herpes genitalis (Whitley and Gnann, 1992), yet both viruses can cause serious systemic diseases including herpetic keratitis and viral encephalitis. These latter diseases are of major concern in immunocompromised patients, especially those undergoing transplant surgery and chemotherapy. Globally, neonatal HSV infection is estimated to occur in about ten cases per 100,000 live births and results in significant morbidity and mortality (Looker et al., 2017). Recent studies also suggest an increased risk for Alzheimer’s disease for patients with the apolipoprotein 4 (APOE-ε4) allele isoform whom are predisposed to an elevated HSV-1 viral load in neural tissue (Steel and Eslick, 2015; Olsson et al., 2016; Agostini et al., 2018; Ashraf et al., 2018; Fulop et al., 2018; Hogestyn et al., 2018; Kristen et al., 2018).

The genus *Varicellovirus* contains the species *human alphaherpesvirus* 3, commonly known as varicella zoster virus (VZV or HHV-3). VZV infection typically occurs at a young age resulting in varicella (chickenpox). Varicella occurs primarily in unvaccinated children and young adults and can lead to complications such as encephalitis, pneumonia, or bronchitis (Gershon et al., 2015). VZV can reactivate later in life to cause neurologic conditions, especially herpes zoster (shingles) and post-herpetic neuralgia (Gilden, 2015). In some individuals, pain remaining after resolution of zoster lesions may become a debilitating disease. Additionally, as one of the TORCH infections, primary VZV infection during pregnancy can cause congenital varicella syndrome consisting of fetal limb hypoplasia, cutaneous scarring, and blindness in the fetus (Ahn et al., 2016). VZV remains the only herpesvirus for which vaccines exist: Varivax (Merck) and ProQuad (Merck) for primary varicella (chickenpox), and Zostavax (Merck) and Shingrix (GlaxoSmithKline) for zoster (shingles). However, these vaccines are not approved for use in pregnant women, people with certain allergies, and those with immune suppression. The vaccine strain can also establish latency and reactivate. The overall disease burden has been significantly reduced as vaccination rates have increased. Breakthrough cases with mild symptoms are occasionally observed in vaccinated persons. Leung et al. (2019) reported in 2019 that “levels of varicella vaccination coverage with two or more doses and the proportion of adolescents with evidence of immunity increased from 2007 to 2014, though 16% lacked evidence of immunity in 2014.” Hence, suboptimal vaccination rates, shingles in the aging population, and the potential role of VZV in other serious diseases such as ocular involvement (Kedar et al., 2019) or vasculopathy (Nagel et al., 2017; Nagel and Bubak, 2018) warrant continued development of effective treatment options for VZV.

Within the *Herpesviridae* sub-family of *Betaherpesvirinae* are the species human cytomegalovirus (HCMV or HHV-5) and human herpesviruses type 6A (HHV-6A), 6B (HHV-6B), and 7 (HHV-7). Shared characteristics include infection and establishment of latency in lymphocytes and monocytes/macrophages. Like the other herpesviruses, most people are infected with the *Betaherpesvirinae* by adulthood, and many do not present with symptoms. Primary infections are typically asymptomatic in immunocompetent individuals, however, HCMV infection during pregnancy can significantly impair fetal development (Zavattoni et al., 2014). HCMV-associated disease pathologies seen in immunocompetent patients include mononucleosis syndrome, diabetes mellitus types 1 and 2, Guillain-Barré syndrome, and potential malignancies [i.e., glioblastoma (Rahman et al., 2019)]. Congenital HCMV is the leading infectious cause of intellectual disability and deafness in children. There are no approved treatments for the ∼0.7% of pregnant women who develop primary HCMV infection during pregnancy (Kenneson and Cannon, 2007; Leruez-Ville et al., 2016). However, there is some evidence that hyperimmune globulin may be beneficial for managing first-trimester infections (Nigro et al., 2019; Schleiss, 2019). Current best practice consists of counseling mothers to limit behaviors that increase risk for contracting a primary HCMV infection during pregnancy. In immunocompromised patients, HCMV is recognized to cause hepatitis, retinitis, colitis, pneumonitis, esophagitis, polyradiculopathy, transverse myelitis, and subacute encephalitis (Gupta and Shorman, 2019). Solid organ transplant recipients often take medications that suppress their T-cell mediated immune response long-term to prevent allograft rejection, and thus are particularly at risk for HCMV disease (Azevedo et al., 2015). The recent approval of letermovir offers a new treatment option for HCMV in allogeneic stem cell transplant patients (see below). Acute HHV-6b and HHV-7 infection, known as 6th disease or roseola infantum, can present as a high fever with accompanying rash in young infants and toddlers. Reactivation of betaherpesviruses is not uncommon in immunocompromised transplant, cancer and AIDS patients (Campadelli-Fiume et al., 1999; Nogalski et al., 2014).

The gammaherpesviruses include Epstein-Barr virus (EBV) and HHV-8. Infectious mononucleosis and Burkitt’s lymphoma result from infection of B-cells with EBV (Houldcroft and Kellam, 2015). The presence of HHV-8, a common opportunistic pathogen of AIDS, is strongly associated with the development of Kaposi’s sarcoma (KS) (Cesarman, 2014).

## Viral Terminase Inhibitors for Treating Human Cytomegalovirus Infections

Toxicity is an issue among approved HCMV therapies including ganciclovir (GCV), foscarnet and cidofovir. These drugs have complex administration and monitoring requirements and are associated with several toxicities including myelosuppression. For example, GCV, the preferred drug of the three is not recommended for patients when the absolute neutrophil count is under 500 cells/ml or when the platelet count is under 25,000/ml (Grayson, 2010). Hematologic malignancies and advanced HIV/AIDS, as well as many of the pharmacotherapies used to treat them, often result in myelosuppression. Patients affected by these diseases are among the highest risk group for severe HCMV infection, yet treatments options are limited. Herpesvirus terminases are part of the molecular nano-motor that initiates viral DNA translocation into empty capsids (Rao and Feiss, 2015). Inhibition of terminase activity results in the accumulation of empty viral capsids and no infectious viral particles are assembled. Terminase has been recognized as a viable antiviral target and several compounds that block the terminase complex have been described including the naphthalenesulfonate BAY 38-4766 (Buerger et al., 2001), benzimidazoles (Underwood et al., 1998; Buerger et al., 2001; Hwang et al., 2007), hydroxypyridonecarboxylic acid compounds (Wang et al., 2017; Kankanala et al., 2018) and letermovir (Goldner et al., 2011; Foolad et al., 2018; Gentry et al., 2019). Letermovir is a dihydroquinazolinyl-acetic acid that represents a new class of HCMV inhibitors that was recently approved for clinical use (Table 1). Briefly, In 2011 Goldner et al. (2011) reported a small molecule compound (AIC246) that had potent *in vitro* activity against HCMV with novel mechanism of action. The compound was active against laboratory and clinical isolates (EC_50_ ∼4 nM with CC_50_ 90 μM). Due to letermovir’s distinct mechanism of action, cross resistance to cidofovir, foscarnet and/or ganciclovir was absent.

**TABLE 1:** Drugs and compounds targeting herpesviral DNA encapsidation and viral capsid assembly.

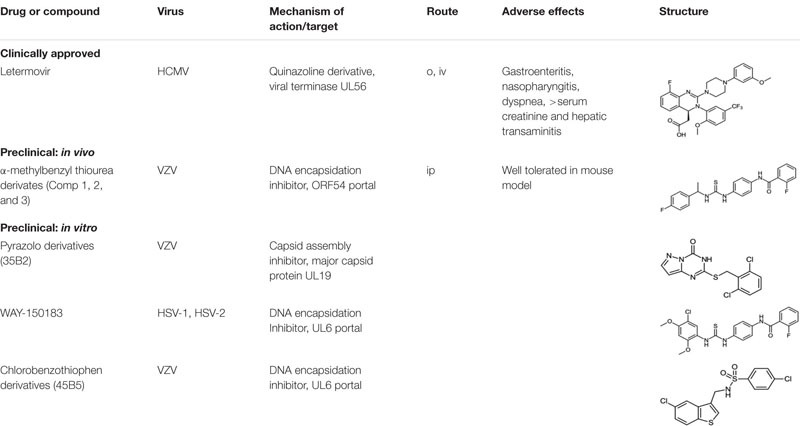

In murine studies of HCMV infected xenografts, AIC246 had favorable toxicological and pharmacological profiles and proved more effective against HCMV than valganciclovir (Lischka et al., 2010). Phase I and II trials demonstrated that AIC246 had a favorable pharmacokinetic profile, was well tolerated and safe (Chemaly et al., 2014; Stoelben et al., 2014; Kropeit et al., 2017a,b, 2018). In a phase three study, letermovir reduced clinically significant HCMV infection in allogeneic stem cell transplant patients (Marty et al., 2017). Successful Phase II and III clinical trials resulted in approval of letermovir in the United States, Canada, Japan, Switzerland, and the European Commission for the prevention of HCMV infection and disease in adult HCMV-seropositive recipients of an allogeneic hematopoietic stem cell transplant (Maffini et al., 2016; Fuji et al., 2017; Chen et al., 2018; Cho et al., 2018; El Helou and Razonable, 2019; Katayama and Iwato, 2019; Ljungman et al., 2019; Mori, 2019).

The viral terminase complex has a low frequency of natural polymorphisms and thus pre-existing mutations associated with resistance in clinical isolates without prior exposure to letermovir seemed unlikely (Pilorge et al., 2014). Douglas et al. (2019) demonstrated that patients taking letermovir had no significant increase in genomic HCMV variants after 24 weeks compared to patients who had been taking a placebo, suggesting low rates of resistance development to letermovir. However, laboratory and clinical resistance to letermovir has been demonstrated and can be mediated by amino acid substitutions within the HCMV UL56 gene (Chou, 2015, 2017; Goldner et al., 2015; Chou et al., 2018; Douglas et al., 2019; Frietsch et al., 2019; Jo et al., 2019; Komatsu et al., 2019; Piret and Boivin, 2019). Studies comparing the development of resistance *in vitro* for GCV and letermovir showed that mutations conferring resistance to letermovir developed more rapidly than gangciclovir (Chou et al., 2018). An increasing number of letermovir resistant isolates have been documented in patients thus complicating clinical management (Razonable, 2018). The emergence of clinical resistance further emphasizes the need for additional anti-HCMV therapeutic options.

## Small Molecule Compounds Targeting *Alphaherpesvirus* Capsids and DNA Encapsidation

### Capsid Assembly Inhibitors

Numerous capsid assembly inhibitors have been described for dengue (Scaturro et al., 2014), Hepatitis C (Kota et al., 2012), and, a first-in-human trial of GSL4, a Hepatitis B virus capsid assembly inhibitor (Merlini et al., 2019). In 2012, the first herpesvirus capsid assembly inhibitor, 35B2, a pyrazolo [1,5-c]1,3,5-triazin-4-one derivative (35B2) (Table 1) was identified (Inoue et al., 2012). 35B2 showed *in vitro* activity against both ACV resistant and sensitive strains of VZV. Strains resistant to 35B2 were found to have mutations in ORF40, the VZV major capsid protein. Infected fibroblasts treated with 35B2 showed altered localization of MCP. Additionally, electron microscopic studies demonstrated the lack of capsid formation in the presence of 35B2 suggesting that the pyrazolo compound affected normal capsid assembly. No *in vivo* results have been reported to date. The data suggest that novel antivirals can be identified that target herpesviral M to inhibit normal capsid formation.

### Portal Inhibitors

Herpesvirus DNA enters and exits the capsid through its portal. The portal protein, located at a single capsid vertex is required for DNA packaging in dsDNA bacteriophages and herpesviruses. Portal proteins of the human herpesviruses, although only modestly conserved at the primary amino acid level, share a conserved core structure.

In 2000, the thiourea compound its N-(4-[3-(5-Chloro-2,4-dimethoxyphenyl)-thioureido]-phenyl)-acetamide and its 2-fluoro-benzamide derivative (WAY-150183; Table 1), were found to inhibit HSV-1 replication *in vitro* (van Zeijl et al., 2000). Lack of a functional portal on the capsid vertex prevented both the cleavage of concatameric viral DNA into genome length progeny and the packaging of the DNA into capsids. In 2003, a class of non-nucleoside N-(α methylbenzyl-N′-arylthiourea analogues, termed Compounds 1, 2, and 3 demonstrated in vitro inhibition of VZV replication (Visalli et al., 2003). The isolation of resistant isolates to either the HSV-1 or VZV inhibitors suggested that the compounds targeted UL6 or ORF54 portal proteins, respectively, and prevented DNA encapsidation (Visalli and van Zeijl, 2003; Visalli et al., 2003, 2014; Di Grandi et al., 2004). The HSV-1 compounds had good *in vitro* activity (0.4–1.5 uM) but no *in vivo* studies were reported for the compound series. The VZV compound series had excellent *in vitro* activity (IC_50_ 10 nM – 1 μM), showed no cellular cytotoxicity (CC_50_ > 37 μM) and was effective against a panel of clinical isolates and ACV-resistant VZV strains. Only empty capsids were observed in the nuclei of VZV infected cells In the presence of compound.

Combined, the results suggested that small molecular thiourea compounds targeting herpesvirus portal proteins represented a new class of anti-herpesvirus inhibitors. Portal proteins are cyclical multimers formed of 12 or 13 subunits that create a hollow empty core or channel. Hence, the thiourea compounds likely inhibit encapsidation by affecting portal structure, function, and/or protein-protein interaction(s). Below, we provide the first *in vivo* evidence that a small molecule compound targeting a viral portal is a viable drug candidate. The host of range of VZV is limited to humans, therefor the *in vivo* efficacy of a thiourea analog was tested in a previously described SCID-Hu VZV infection model (Rowe et al., 2010; De et al., 2014). The results presented here are the first for any compound targeting a viral portal in an animal model.

**FIGURE 1:**
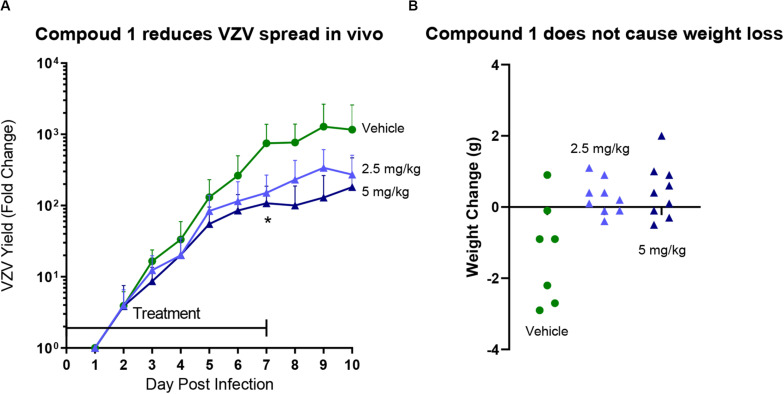
SCID-Hu mice with thymus/liver xenografts were inoculated with VZV_LUC_ (firefly luciferase) and treated with vehicle or Comp 1 at 2.5 or 5 mg/kg in Cremaphor-DMSO-Saline s.c. daily from 2 h after infection to Day 7. Each mouse was weighed on the day before virus inoculation in order to calculate the drug concentration in the preparations. **(A)** Mice were injected with D-luciferin (150 mg/kg s.c.) and then VZV replication was measured by IVIS bioluminescence imaging daily from days 1–10. VZV yield was calculated as fold change for each mouse by dividing Total Flux values by the lowest value on Day 1. The lines are the average for the group (*N* = 7 Vehicle group, *N* = 8 treatment groups), the error bars are the standard deviation of the mean. Both doses of Comp 1 significantly reduced VZV yield compared to vehicle on Day 7 (*p* = 0.0037; 1-way ANOVA with Dunnett’s Multiple Comparison Test). **(B)** Mice were weighed on Day 10 and the net change for each mouse was calculated. Each symbol represents one mouse (some mice in the Vehicle group lost weight due aggressive activity). No mice died in any of the three groups.

Comp 1 (Table 1), an α-methylbenzyl thiorurea compound that prevented VZV DNA encapsidation *in vitro*, was evaluated in a SCID-Hu thymus/liver VZV infection model (Figure 1). Both doses of Comp 1 significantly reduced the VZV growth rate compared to vehicle (*p* = 0.0037). The treatment phase was extended beyond the typical 7 days to observe any potential side effects of the compound. Only the vehicle group lost weight whereas the compound treatment groups showed positive weight gain and the mice appeared well-hydrated with smooth fur indicating that Comp 1 was well-tolerated. Previous studies showed that compounds from the α-methylbenzyl series were not toxic in multiple cell lines [(Visalli et al., 2003), data not shown] nor in murine, canine and primate pharmacokinetic studies (data not shown). The results presented here merit further investigation of α-methyl benzyl analogs, in particular, newer compounds that have been optimized for oral bioavailability and that have activity against other herpesvirus family members.

In 2017, a 5-chlorobenzo[b]thiophen derivative (45B5) (Table 1) was characterized as a potential anti-VZV compound (EC_50_ 16.9 μM) (Yasui et al., 2017). All 45B5 resistant laboratory strains were found to have at least one mutation in ORF54. Cells infected in the presence of compound had decreased viral DNA synthesis and did not appear to affect portal-scaffold protein interactions. Therefore, it was postulated that 45B5 inhibits viral DNA release from the capsid portal vertex at the nuclear pore.

## Perspective on Future Directions

The *Herpesviridae* are known to contribute significantly to worldwide human and animal morbidity and mortality. The herpesviral double stranded DNA genome is encased in an icosahedral protein capsid that is tethered to a lipid bilayer envelope by viral tegument proteins. The family name is derived from the Greek word *herpein* meaning “to creep” in reference to the unifying feature of these viruses to establish latency. Herpesvirus pathogenesis and disease manifestations are determined by the cell and tissue types that can be infected by the different viral species.

Nucleoside analogs such as ACV have been indispensable prophylactic and therapeutic treatment options. Even with the availability of effective nucleoside analogs, human herpesviruses are associated with substantial morbidity and mortality. We and others have proposed the development of new agents with unique mechanisms of action such as targeting the viral helicase-primase, ribonucleotide reductase, protease, glycoprotein attachment and fusion, viral protein kinases, and DNA encapsidation (e.g., terminase and portal inhibitors). Although several candidates with novel targets advanced to phase II and III clinical trials, only one compound, letermovir, has been approved for human use since the antisense antiviral fomivirsen (HCMV retinitis) and famciclovir more than twenty years ago (1998). The recent FDA approval of the letermovir is proof of principle that targeting herpesvirus DNA encapsidation is a viable chemotherapeutic strategy. In Figure 1 we show for the first time that targeting the viral portal protein *in vivo* is a viable option. It is possible that new compounds can be identified that target any of the three herpesvirus terminase subunits and/or portal proteins. There are still no approved chemotherapeutic options for EBV or HHV-6, 7, and 8. The limitations of current therapies due to specificity, toxicity, bioavailability and resistance merit the continued discovery and research of novel herpesvirus antivirals.

## Data Availability Statement

The datasets generated for this study are available on request to the corresponding author.

## Ethics Statement

The animal study was reviewed and approved by the IACUC [formerly known as the Committee for the Humane Use of Animals (CHUA)] evaluates the animal care and use program at SUNY Upstate Medical University and facilitates the conduct of meaningful scientific research that: (1) avoids or minimizes discomfort, distress, and pain in experimental animals consistent with sound scientific practices, (2) uses the minimum number of animals necessary to obtain valid results, and (3) considers non-animal models wherever possible. OLAW PHS Assurance: D16-00318; USDA Certificate: 21-R-0037; NYSDOH Unit: A073; AAALAC: Full Accreditation (accredited since 7/31/1999).

## Author Contributions

TK, JM, and RV wrote the manuscript. JM and RV designed the experiments and analyzed the data. DL, ML, WC, and JM performed the animal studies. All authors contributed to the article and approved the submitted version.

## Conflict of Interest

The authors declare that the research was conducted in the absence of any commercial or financial relationships that could be construed as a potential conflict of interest.

## References

[B1] Agostini S. Mancuso R. Hernis A. Costa A. S. Nemni R. Clerici M. (2018). HSV-1-Specific IgG subclasses distribution and serum neutralizing activity in Alzheimer’s disease and in mild cognitive impairment. *J. Alzheimers Dis.* 63 131–138. 10.3233/JAD-170966 29578484

[B2] Ahn K. H. Park Y. J. Hong S. C. Lee E. H. Lee J. S. Oh M. J. (2016). Congenital varicella syndrome: a systematic review. *J. Obstetr. Gynaecol.* 36 563–566. 10.3109/01443615.2015.1127905 26965725

[B3] Arvin A. M. (2007). *Human Herpesviruses: Biology, Therapy, and Immunoprophylaxis.* Cambridge: Cambridge University Press, 1388. 21348071

[B4] Ashraf G. M. Tarasov V. V. Makhmutovsmall A. C. A. Chubarev V. N. Avila-Rodriguez M. Bachurin S. O. (2018). The possibility of an infectious etiology of Alzheimer disease. *Mol. Neurobiol.* 56 4479–4491. 10.1007/s12035-018-1388-y 30338482

[B5] Azevedo L. S. Pierrotti L. C. Abdala E. Costa S. F. Strabelli T. M. Campos S. V. (2015). Cytomegalovirus infection in transplant recipients. *Clinics* 70 515–523. 10.6061/clinics/2015(07)09 26222822 PMC4496754

[B6] Buerger I. Reefschlaeger J. Bender W. Eckenberg P. Popp A. Weber O. (2001). A novel nonnucleoside inhibitor specifically targets cytomegalovirus DNA maturation via the UL89 and UL56 gene products. *J. Virol.* 75 9077–9086. 10.1128/JVI.75.19.9077-9086.2001 11533171 PMC114476

[B7] Campadelli-Fiume G. Mirandola P. Menotti L. (1999). Human herpesvirus 6: an emerging pathogen. *Emerg. Infect. Dis.* 5 353–366. 10.3201/eid0503.990306 10341172 PMC2640789

[B8] Cesarman E. (2014). Gammaherpesviruses and lymphoproliferative disorders. *Annu. Rev. Pathol.* 9 349–372. 10.1146/annurev-pathol-012513-104656 24111911

[B9] Chemaly R. F. Ullmann A. J. Stoelben S. Richard M. P. Bornhauser M. Groth C. (2014). Letermovir for cytomegalovirus prophylaxis in hematopoietic-cell transplantation. *N. Engl. J. Med.* 370 1781–1789. 10.1056/NEJMoa1309533 24806159

[B10] Chen K. Cheng M. P. Hammond S. P. Einsele H. Marty F. M. (2018). Antiviral prophylaxis for cytomegalovirus infection in allogeneic hematopoietic cell transplantation. *Blood Adv.* 2 2159–2175. 10.1182/bloodadvances.2018016493 30154125 PMC6113617

[B11] Cho J. C. Le A. D. Locke S. C. (2018). Letermovir for prophylaxis of cytomegalovirus in allogeneic hematopoietic stem cell recipients. *Drugs Today* 54 361–368. 10.1358/dot.2018.54.6.2833982 29998227

[B12] Chou S. (2015). Rapid in vitro evolution of human cytomegalovirus UL56 mutations that confer letermovir resistance. *Antimicrob. Agents Chemother.* 59 6588–6593. 10.1128/AAC.01623-15 26259791 PMC4576131

[B13] Chou S. (2017). A third component of the human cytomegalovirus terminase complex is involved in letermovir resistance. *Antiviral Res.* 148 1–4. 10.1016/j.antiviral.2017.10.019 29107686 PMC5687998

[B14] Chou S. Satterwhite L. E. Ercolani R. J. (2018). New Locus of Drug Resistance in the Human Cytomegalovirus UL56 Gene Revealed by In Vitro Exposure to Letermovir and Ganciclovir. *Antimicrob. Agents Chemother.* 62 10.1128/AAC.00922-18 29914965 PMC6125535

[B15] De C. Liu D. Zheng B. Singh U. S. Chavre S. White C. (2014). beta-l-1-[5-(E-2-bromovinyl)-2-(hydroxymethyl)-1,3-(dioxolan-4-yl)] uracil (l-BHDU) prevents varicella-zoster virus replication in a SCID-Hu mouse model and does not interfere with 5-fluorouracil catabolism. *Antiviral Res.* 110 10–19. 10.1016/j.antiviral.2014.07.007 25051026 PMC4171207

[B16] Di Grandi M. J. Curran K. J. Feigelson G. Prashad A. Ross A. A. Visalli R. (2004). Thiourea inhibitors of herpesviruses. Part 3: inhibitors of varicella zoster virus. *Bioorg. Med. Chem. Lett.* 14 4157–4160. 10.1016/j.bmcl.2004.06.025 15261261

[B17] Douglas C. M. Barnard R. Holder D. Leavitt R. Levitan D. Maguire M. (2019). Letermovir resistance analysis in a clinical trial of cytomegalovirus prophylaxis for hematopoietic stem cell transplant recipients. *J. Infect. Dis.* 221 1117–1126. 10.1093/infdis/jiz577 31781762 PMC7075417

[B18] El Helou G. Razonable R. R. (2019). Letermovir for the prevention of cytomegalovirus infection and disease in transplant recipients: an evidence-based review. *Infect. Drug Resist.* 12 1481–1491. 10.2147/IDR.S180908 31239725 PMC6556539

[B19] Foolad F. Aitken S. L. Chemaly R. F. (2018). Letermovir for the prevention of cytomegalovirus infection in adult cytomegalovirus-seropositive hematopoietic stem cell transplant recipients. *Expert Rev. Clin. Pharmacol.* 11 931–941. 10.1080/17512433.2018.1500897 30004790

[B20] Frietsch J. J. Michel D. Stamminger T. Hunstig F. Birndt S. Schnetzke U. (2019). In vivo emergence of UL56 C325Y cytomegalovirus resistance to letermovir in a patient with acute myeloid leukemia after hematopoietic cell transplantation. *Mediterr. J. Hematol. Infect. Dis.* 11:e2019001. 10.4084/MJHID.2019.001 30671207 PMC6328044

[B21] Fuji S. Einsele H. Kapp M. (2017). Cytomegalovirus disease in hematopoietic stem cell transplant patients: current and future therapeutic options. *Curr. Opin. Infect. Dis.* 30 372–376. 10.1097/QCO.0000000000000375 28505028

[B22] Fulop T. Itzhaki R. F. Balin B. J. Miklossy J. Barron A. E. (2018). Role of microbes in the development of Alzheimer’s disease: state of the art - an international symposium presented at the 2017 IAGG Congress in San Francisco. *Front. Genet.* 9:362. 10.3389/fgene.2018.00362 30250480 PMC6139345

[B23] Gentry B. G. Bogner E. Drach J. C. (2019). Targeting the terminase: an important step forward in the treatment and prophylaxis of human cytomegalovirus infections. *Antiviral Res.* 161 116–124. 10.1016/j.antiviral.2018.11.005 30472161

[B24] Gershon A. A. Breuer J. Cohen J. I. Cohrs R. J. Gershon M. D. Gilden D. (2015). Varicella zoster virus infection. *Nat. Rev. Dis. Prim.* 1:15016. 10.1038/nrdp.2015.16 27188665 PMC5381807

[B25] Gilden D. (2015). Varicella-zoster virus infections. *Continuum* 21 1692–1703. 10.1212/CON.0000000000000246 26633783

[B26] Goldner T. Hewlett G. Ettischer N. Ruebsamen-Schaeff H. Zimmermann H. Lischka P. (2011). The novel anticytomegalovirus compound AIC246 (Letermovir) inhibits human cytomegalovirus replication through a specific antiviral mechanism that involves the viral terminase. *J. Virol.* 85 10884–10893. 10.1128/JVI.05265-11 21752907 PMC3187482

[B27] Goldner T. Zimmermann H. Lischka P. (2015). Phenotypic characterization of two naturally occurring human Cytomegalovirus sequence polymorphisms located in a distinct region of ORF UL56 known to be involved in in vitro resistance to letermovir. *Antiviral Res.* 116 48–50. 10.1016/j.antiviral.2015.01.006 25637709

[B28] Grayson M. L. (2010). *Kucers’ the Use of Antibiotics : A Clinical Review of Antibacterial, Antifungal, Antiparasitic and Antiviral Drugs*, Seventh Edn Boca Raton, FL: CRC Press.

[B29] Groves M. J. (2016). Genital herpes: a review. *Am. Fam. Physician* 93 928–934. 27281837

[B30] Gupta M. Shorman M. (2019). *Cytomegalovirus.* Treasure Island, FL: StatPearls.

[B31] Hogestyn J. M. Mock D. J. Mayer-Proschel M. (2018). Contributions of neurotropic human herpesviruses herpes simplex virus 1 and human herpesvirus 6 to neurodegenerative disease pathology. *Neural Regen. Res.* 13 211–221. 10.4103/1673-5374.226380 29557362 PMC5879884

[B32] Houldcroft C. J. Kellam P. (2015). Host genetics of Epstein-Barr virus infection, latency and disease. *Rev. Med. Virol.* 25 71–84. 10.1002/rmv.1816 25430668 PMC4407908

[B33] Hwang J. S. Kregler O. Schilf R. Bannert N. Drach J. C. Townsend L. B. (2007). Identification of acetylated, tetrahalogenated benzimidazole D-ribonucleosides with enhanced activity against human cytomegalovirus. *J. Virol.* 81 11604–11611. 10.1128/JVI.01130-07 17728228 PMC2168816

[B34] Inoue N. Matsushita M. Fukui Y. Yamada S. Tsuda M. Higashi C. (2012). Identification of a varicella-zoster virus replication inhibitor that blocks capsid assembly by interacting with the floor domain of the major capsid protein. *J. Virol.* 86 12198–12207. 10.1128/JVI.01280-12 22933294 PMC3486443

[B35] Jo H. Kwon D. E. Han S. H. Min S. Y. Hong Y. M. Lim B. J. (2019). *De novo* genotypic heterogeneity in the UL56 region in cytomegalovirus-infected tissues: implications for primary Letermovir Resistance. *J. Infect. Dis.* 221 1480–1487. 10.1093/infdis/jiz642 31802131

[B36] Kankanala J. Wang Y. Geraghty R. J. Wang Z. (2018). Hydroxypyridonecarboxylic acids as inhibitors of human cytomegalovirus pUL89 Endonuclease. *ChemMedChem* 13 1658–1663. 10.1002/cmdc.201800283 29968426

[B37] Katayama Y. Iwato K. (2019). [Cytomegalovirus infections following allogeneic hematopoietic stem cell transplantation: prophylaxis and treatment]. *Rinsho Ketsueki* 60 635–645. 10.11406/rinketsu.60.635 31281156

[B38] Kedar S. Jayagopal L. N. Berger J. R. (2019). Neurological and ophthalmological manifestations of Varicella Zoster Virus. *J. Neuroophthalmol.* 39 220–231. 10.1097/WNO.0000000000000721 30188405

[B39] Kenneson A. Cannon M. J. (2007). Review and meta-analysis of the epidemiology of congenital cytomegalovirus (CMV) infection. *Rev. Med. Virol.* 17 253–276. 10.1002/rmv.535 17579921

[B40] Komatsu T. E. Hodowanec A. C. Colberg-Poley A. M. Pikis A. Singer M. E. O’Rear J. J. (2019). In-depth genomic analyses identified novel letermovir resistance-associated substitutions in the cytomegalovirus UL56 and UL89 gene products. *Antiviral Res.* 169:104549. 10.1016/j.antiviral.2019.104549 31279814

[B41] Kota S. Takahashi V. Ni F. Snyder J. K. Strosberg A. D. (2012). Direct binding of a hepatitis C virus inhibitor to the viral capsid protein. *PLoS One* 7:e32207. 10.1371/journal.pone.0032207 22389688 PMC3289641

[B42] Kristen H. Sastre I. Munoz-Galdeano T. Recuero M. Aldudo J. Bullido M. J. (2018). The lysosome system is severely impaired in a cellular model of neurodegeneration induced by HSV-1 and oxidative stress. *Neurobiol. Aging* 68 5–17. 10.1016/j.neurobiolaging.2018.03.025 29689425

[B43] Kropeit D. McCormick D. Erb-Zohar K. Moiseev V. S. Kobalava Z. D. Stobernack H. P. (2017a). Pharmacokinetics and safety of the anti-human cytomegalovirus drug letermovir in subjects with hepatic impairment. *Br. J. Clin. Pharmacol.* 83 2678–2686. 10.1111/bcp.13376 28722153 PMC5698571

[B44] Kropeit D. Scheuenpflug J. Erb-Zohar K. Halabi A. Stobernack H. P. Hulskotte E. G. J. (2017b). Pharmacokinetics and safety of letermovir, a novel anti-human cytomegalovirus drug, in patients with renal impairment. *Br. J. Clin. Pharmacol.* 83 1944–1953. 10.1111/bcp.13292 28345163 PMC5555856

[B45] Kropeit D. von Richter O. Stobernack H. P. Rubsamen-Schaeff H. Zimmermann H. (2018). Pharmacokinetics and safety of letermovir coadministered with cyclosporine A or tacrolimus in healthy subjects. *Clin. Pharmacol. Drug Dev.* 7 9–21. 10.1002/cpdd.388 28967706

[B46] Leruez-Ville M. Ghout I. Bussieres L. Stirnemann J. Magny J. F. Couderc S. (2016). In utero treatment of congenital cytomegalovirus infection with valacyclovir in a multicenter, open-label, phase II study. *Am. J. Obstetr. Gynecol.* 215 462.e1–462.e10. 10.1016/j.ajog.2016.04.003 27083761

[B47] Leung J. Reagan-Steiner S. Lopez A. Jeyarajah J. Marin M. (2019). Varicella vaccination among US adolescents: coverage and missed opportunities, 2007-2014. *J. Public Health Manage. Pract.* 25 E19–E26. 10.1097/PHH.0000000000000819 29889179 PMC6286230

[B48] Lischka P. Hewlett G. Wunberg T. Baumeister J. Paulsen D. Goldner T. (2010). *In vitro* and *in vivo* activities of the novel anticytomegalovirus compound AIC246. *Antimicrob. Agents Chemother.* 54 1290–1297. 10.1128/AAC.01596-09 20047911 PMC2826024

[B49] Ljungman P. de la Camara R. Robin C. Crocchiolo R. Einsele H. Hill J. A. (2019). Guidelines for the management of cytomegalovirus infection in patients with haematological malignancies and after stem cell transplantation from the 2017 European Conference on Infections in Leukaemia (ECIL 7). *Lancet Infect. Dis.* 19 e260–e272. 10.1016/S1473-3099(19)30107-0 31153807

[B50] Looker K. J. Magaret A. S. May M. T. Turner K. M. Vickerman P. Gottlieb S. L. (2015a). Global and regional estimates of prevalent and incident herpes simplex virus Type 1 infections in 2012. *PLoS One* 10:e0140765. 10.1371/journal.pone.0140765 26510007 PMC4624804

[B51] Looker K. J. Magaret A. S. May M. T. Turner K. M. E. Vickerman P. Newman L. M. (2017). First estimates of the global and regional incidence of neonatal herpes infection. *Lancet Global Health* 5 e300–e309. 10.1016/S2214-109X(16)30362-X 28153513 PMC5837040

[B52] Looker K. J. Magaret A. S. Turner K. M. Vickerman P. Gottlieb S. L. Newman L. M. (2015b). Global estimates of prevalent and incident herpes simplex virus type 2 infections in 2012. *PLoS One* 10:e114989. 10.1371/journal.pone.0114989 25608026 PMC4301914

[B53] Maffini E. Giaccone L. Festuccia M. Brunello L. Busca A. Bruno B. (2016). Treatment of CMV infection after allogeneic hematopoietic stem cell transplantation. *Expert Rev. Hematol.* 9 585–596. 10.1080/17474086.2016.1174571 27043241

[B54] Marty F. M. Ljungman P. Chemaly R. F. Maertens J. Dadwal S. S. Duarte R. F. (2017). Letermovir prophylaxis for cytomegalovirus in hematopoietic-cell transplantation. *N. Engl. J. Med.* 377 2433–2444. 10.1056/NEJMoa1706640 29211658

[B55] Merlini L. Sabatelli P. Antoniel M. Carinci V. Niro F. Monetti G. (2019). Congenital myopathy with hanging big toe due to homozygous myopalladin (MYPN) mutation. *Skelet. Muscle* 9:14. 10.1186/s13395-019-0199-9 31133047 PMC6535860

[B56] Mori T. (2019). [Management of cytomegalovirus infection after hematopoietic stem cell transplantation]. *Rinsho Ketsueki* 60 1337–1340. 10.11406/rinketsu.60.1337 31597861

[B57] Nagel M. A. Bubak A. N. (2018). Varicella Zoster Virus Vasculopathy. *J. Infect. Dis.* 218(Suppl._2), S107–S112. 10.1093/infdis/jiy425 30247600 PMC6151079

[B58] Nagel M. A. Jones D. Wyborny A. (2017). Varicella zoster virus vasculopathy: the expanding clinical spectrum and pathogenesis. *J. Neuroimmunol.* 308 112–117. 10.1016/j.jneuroim.2017.03.014 28335992 PMC5489071

[B59] Nigro G. Adler S. P. Congenital Cytomegalic Disease Collaborating Group (2019). High-dose CMV hyperimmune globulin (HIG) and maternal CMV DNAemia independently predict infant outcome in pregnant women with a primary cytomegalovirus (CMV) infection. *Clin. Infect. Dis.* 10.1093/cid/ciz1030 [Epub ahead of print]. 31628849

[B60] Nogalski M. T. Collins-McMillen D. Yurochko A. D. (2014). Overview of human cytomegalovirus pathogenesis. *Methods Mol. Biol.* 1119 15–28. 10.1007/978-1-62703-788-4_2 24639215

[B61] Olsson J. Lovheim H. Honkala E. Karhunen P. J. Elgh F. Kok E. H. (2016). HSV presence in brains of individuals without dementia: the TASTY brain series. *Dis. Models Mech.* 9 1349–1355. 10.1242/dmm.026674 27664135 PMC5117234

[B62] Pilorge L. Burrel S. Ait-Arkoub Z. Agut H. Boutolleau D. (2014). Human cytomegalovirus (CMV) susceptibility to currently approved antiviral drugs does not impact on CMV terminase complex polymorphism. *Antiviral Res.* 111 8–12. 10.1016/j.antiviral.2014.08.014 25194992

[B63] Piret J. Boivin G. (2019). Clinical development of letermovir and maribavir: overview of human cytomegalovirus drug resistance. *Antiviral Res.* 163 91–105. 10.1016/j.antiviral.2019.01.011 30690043

[B64] Rahman M. Dastmalchi F. Karachi A. Mitchell D. (2019). The role of CMV in glioblastoma and implications for immunotherapeutic strategies. *Oncoimmunology* 8:e1514921. 10.1080/2162402X.2018.1514921 30546954 PMC6287786

[B65] Rao V. B. Feiss M. (2015). Mechanisms of DNA packaging by large double-stranded DNA viruses. *Annu. Rev. Virol.* 2 351–378. 10.1146/annurev-virology-100114-055212 26958920 PMC4785836

[B66] Razonable R. R. (2018). Drug-resistant cytomegalovirus: clinical implications of specific mutations. *Curr. Opin. Organ Transplant.* 23 388–394. 10.1097/MOT.0000000000000541 29794552

[B67] Rowe J. Greenblatt R. J. Liu D. Moffat J. F. (2010). Compounds that target host cell proteins prevent varicella-zoster virus replication in culture, ex vivo, and in SCID-Hu mice. *Antiviral Res.* 86 276–285. 10.1016/j.antiviral.2010.03.007 20307580 PMC2866756

[B68] Scaturro P. Trist I. M. Paul D. Kumar A. Acosta E. G. Byrd C. M. (2014). Characterization of the mode of action of a potent dengue virus capsid inhibitor. *J. Virol.* 88 11540–11555. 10.1128/JVI.01745-14 25056895 PMC4178822

[B69] Schleiss M. R. (2019). The value of hyperimmune globulin (HIG) in pregnancies complicated by cytomegalovirus infection: a continuing saga. *Clin. Infect. Dis.* 10.1093/cid/ciz1036 [Epub ahead of print]. 31628841 PMC7486836

[B70] Steel A. J. Eslick G. D. (2015). Herpes viruses increase the risk of Alzheimer’s disease: a meta-analysis. *J. Alzheimers Dis.* 47 351–364. 10.3233/JAD-140822 26401558

[B71] Stoelben S. Arns W. Renders L. Hummel J. Muhlfeld A. Stangl M. (2014). Preemptive treatment of Cytomegalovirus infection in kidney transplant recipients with letermovir: results of a Phase 2a study. *Transplant Int.* 27 77–86. 10.1111/tri.12225 24164420

[B72] Underwood M. R. Harvey R. J. Stanat S. C. Hemphill M. L. Miller T. Drach J. C. (1998). Inhibition of human cytomegalovirus DNA maturation by a benzimidazole ribonucleoside is mediated through the UL89 gene product. *J. Virol.* 72 717–725. 9420278 10.1128/jvi.72.1.717-725.1998 PMC109427

[B73] van Zeijl M. Fairhurst J. Jones T. R. Vernon S. K. Morin J. LaRocque J. (2000). Novel class of thiourea compounds that inhibit herpes simplex virus type 1 DNA cleavage and encapsidation: resistance maps to the UL6 gene. *J. Virol.* 74 9054–9061. 10.1128/jvi.74.19.9054-9061.2000 10982350 PMC102102

[B74] Visalli M. A. House B. L. Selariu A. Zhu H. Visalli R. J. (2014). The varicella-zoster virus portal protein is essential for cleavage and packaging of viral DNA. *J. Virol.* 88 7973–7986. 10.1128/JVI.00376-14 24807720 PMC4097804

[B75] Visalli R. J. Fairhurst J. Srinivas S. Hu W. Feld B. DiGrandi M. (2003). Identification of small molecule compounds that selectively inhibit varicella-zoster virus replication. *J. Virol.* 77 2349–2358. 10.1128/jvi.77.4.2349-2358.2003 12551972 PMC141108

[B76] Visalli R. J. van Zeijl M. (2003). DNA encapsidation as a target for anti-herpesvirus drug therapy. *Antiviral Res.* 59 73–87. 12895691 10.1016/s0166-3542(03)00108-6

[B77] Wang Y. Mao L. Kankanala J. Wang Z. Geraghty R. J. (2017). Inhibition of human cytomegalovirus pUL89 terminase subunit blocks virus replication and genome cleavage. *J. Virol.* 91:e02152-16. 10.1128/JVI.02152-16 27881652 PMC5244350

[B78] Whitley R. J. Gnann J. W. Jr. (1992). Acyclovir: a decade later. *N. Engl. J. Med.* 327 782–789. 10.1056/NEJM199209103271108 1288525

[B79] Yasui R. Yoshida C. Yamaguchi T. Inoue N. (2017). Characterization of an anti-varicella-zoster virus compound that targets the portal protein encoded by ORF54. *Microbiol. Immunol.* 61 398–402. 10.1111/1348-0421.12507 28833387

[B80] Zavattoni M. Lombardi G. Rognoni V. Furione M. Klersy C. Stronati M. (2014). Maternal, fetal, and neonatal parameters for prognosis and counseling of HCMV congenital infection. *J. Med. Virol.* 86 2163–2170. 10.1002/jmv.23954 24777597

